# Autophagy in kidney transplants of sirolimus treated recipients

**DOI:** 10.15171/jnp.2017.15

**Published:** 2016-10-22

**Authors:** Sagar Bhayana, Arpita Baisantry, Thomas D. Kraemer, Christoph Wrede, Jan Hegermann, Jan-Hinrich Bräsen, Clemens Bockmeyer, Jan Ulrich Becker, Matthias Ochs, Wilfried Gwinner, Hermann Haller, Anette Melk, Roland Schmitt

**Affiliations:** ^1^Department of Nephrology and Hypertension, Hannover Medical School, Hannover, Germany; ^2^Department of Paediatric Nephrology and Gastroenterology, Hannover Medical School, Hannover, Germany; ^3^Institute of Functional and Applied Anatomy, Hannover Medical School, Hannover, Germany; ^4^REBIRTH Cluster of Excellence, Hannover, Germany; ^5^Institute of Pathology, Medical School Hannover, Hannover, Germany

**Keywords:** Autophagy, mTOR inhibitors, Sirolimus, Kidney transplant biopsy, Electron microscopy

## Abstract

**Background:**

Mammalian target of rapamycin (mTOR) inhibitors are increasingly used as immunosuppressive agents in kidney transplantation. In the experimental setting it has been shown that mTOR inhibitors promote autophagy, but the concept that this might also occur in transplant patients has not been addressed.

**Objectives:**

This study was designed to investigate the association between mTOR inhibition and autophagy in renal transplants under routine clinical conditions.

**Materials and Methods:**

Protocol transplant biopsies of patients receiving sirolimus were compared to biopsies of patients treated without mTOR inhibitor. Electron microscopy was used for quantitative stereological analysis of autophagosomal volume fractions. Ultrastructural analysis was focused on podocytes to avoid cell type bias. Autophagy-related gene products were profiled by QPCR from laser assisted microdissected glomeruli and by immunohistochemistry for semiquantitative evaluation.

**Results:**

By electron microscopy, we observed a significant > 50% increase in podocytic autophagosomal volume fractions in patients treated with sirolimus. Evaluation of biopsy material from the same patients using transcriptional profiling of laser capture microdissected glomeruli revealed no differences in autophagy-related gene expressions. Immunohistochemical evaluation of autophagic degradation product p62 was also unaltered whereas a significant increase was observed in podocytic LC3 positivity in biopsies of sirolimus treated patients.

**Conclusions:**

These results indicate an association of sirolimus treatment and autophagosome formation in transplant patients. However, they might reflect autophagosomal buildup rather than increased autophagic flux. Further research is needed to investigate the potential functional consequences in short- and long-term outcome of patients treated with mTOR inhibitors.

**Implication for health policy/practice/research/medical education:**

In this proof-of-principle study we found that routine immunosuppressive treatment of kidney allograft recipients with sirolimus was associated with an increased autophagosomal volume fraction in podocytes as shown by electron microscopy. At the same time we found only minor changes in biochemical parameters of autophagy. While it is not possible to conclude from these findings whether mTOR inhibition increased autophagic flux or rather induced autophagosomal buildup, these data provide the first formal evidence for a significant impact of sirolimus on the autophagic pathway in the clinical transplant setting. This link is of practical relevance and should be further investigated given the increasingly recognized role of dysregulated autophagy in kidney disease.

## 1. Background

Autophagy is an evolutionary conserved mechanism that sequesters proteins and damaged organelles within double-membrane vesicles termed autophagosomes, which fuse with lysosomes to degrade their cargo. Thereby autophagy counteracts accumulation of damaged organelles and provides energy (1). Defects in autophagy have been linked to accelerated aging, reduced metabolic fitness, cancer, cardiovascular disease and several other human pathologies (2). Autophagy has been found to protect the kidney from acute tubular injury (3) and it was concluded that pharmaceutical autophagic activation may be beneficial for different diseases (4).

One of the key regulators of autophagy is mammalian target of rapamycin (mTOR). mTOR is a serine-threonine kinase, which interacts with regulatory proteins to form the mTOR complex 1 (mTORC1). mTORC1 functions as a nutrient sensor, which when activated stimulates protein synthesis and cell growth and blocks autophagy. In the experimental setting, pharmaceutical inhibition of mTOR by sirolimus (rapamycin) or its derivative, everolimus, prevents the formation of mTORC1 and thus triggers the initiation of autophagic processes (1). mTOR inhibitors are used as immunosuppressive agents in transplantation medicine as they also block cytokine receptor-dependent signal transduction and thereby inhibit the activation of T lymphocytes (5).

## 2. Objectives

Despite the growing use of mTOR inhibitors there is no data about their potential impact on autophagy in the setting of transplantation. Based on experimental studies, it has been assumed that autophagy might be induced in sirolimus treated patients (6), but this has never been formally tested. It was the goal of this proof-of-principle study to fill this gap by examining renal transplant biopsies using different methodological approaches.

## 3. Patients and Methods

### 3.1. Patient selection and study design

All patients transplanted between 2002 and 2007 were included if they fulfilled the following criteria: transplant protocol biopsy taken at 3 months after transplantation; samples for transmission electron microscopy (TEM) available; routine pathological assessment of the biopsy showed no signs of rejection or acute tubular necrosis; immunosuppressive treatment included sirolimus at the time of biopsy. Protocol biopsies were performed after obtaining a written consent according to our center’s policy, approved by the Hannover Medical School ethics committee. Urine samples were collected at the time of biopsies and patients were excluded if they showed signs of urinary tract infection. Fifteen patients who received sirolimus in combination with calcineurin inhibitors (CNIs) (cyclosporine n = 7; tacrolimus n = 5) and mycophenolate mofetil (MMF) or only with MMF (n = 3) fulfilled the inclusion criteria. As our analyses revealed no differences between sirolimus treatment alone or sirolimus treatment in combination with CNI, data from all 15 sirolimus treated patients were pooled (sirolimus group). Control patients with matching dates of transplantation were selected by the same inclusion criteria as above, when they were treated with cyclosporine and MMF alone (n = 20). Six patients who were treated with belatacept and MMF were included as an additional control group. All patients received anti-interleukin-2 receptor antibody induction, initial methylprednisolone, and prednisolone in the maintenance therapy. Demographical and clinical data were taken from patients charts and are shown in Table 1. Allograft function was assessed by the estimated glomerular filtration rate (eGFR) according to the Cockroft and Gault formula.

### 3.2. Transmission electron microscopy and stereological analysis of autophagosomes

Small samples of the renal cortex from the biopsies were fixed in glutaraldehyde and embedded in Araldit CY212 (PLANO, Wetzlar, Germany) and analyzed with a TEM Morgagni 268 (FEI Company, Eindhoven, NL). Glomeruli from each biopsy were scanned by a blinded investigator who selected podocytes by ultrastructural criteria. Sixty to 80 digital images (1024 × 1024 pixels) per sample were captured in a zig-zag fashion at 22 000X magnification throughout glomeruli with a constant distance between every two images by adjusting the co-ordinates according to principles of systematic uniform random sampling. Autophagosomes were identified using previously established criteria (7). Briefly, vacuoles were classified as autophagosomes when they met with two or more of the following criteria: double membranes (complete or at least partially visible), absence of ribosomes attached to the cytosolic side of the membrane, luminal density similar to cytosol, and identifiable organelles or parts of organelles in their lumens. Autophagosomes were stereologically quantified using a test grid, composed of points (81 points per image) which was superimposed on the digital images using the STEPanizer^©^ program (8). The number of points that were localized on autophagosomes were counted and divided by the total points that were localized on podocytes. The autophagosomal volume fraction within the main cell body of podocytes was calculated for each sample. The number of test points counted on autophagosomes was 80-130 per patient, thus assuring a sufficient precision of the stereological estimates (9).

### 3.3. Laser assisted microdissection and RNA extraction

Serial sections (4-µm-thick) of formalin-fixed, paraffin-embedded biopsies were mounted on poly-L-lysine coated membranes fixed onto a metal frame. Using the CellCut Plus system (MMI Molecular Machines & Industries AG, Glattbrugg, Switzerland), laser-assisted microdissection of glomeruli was performed. A minimum of 15 glomeruli per sample was collected from 3-4 serial sections. The microdissected material was subsequently suspended in digestion buffer (4.2M guanidium thiocyanate, 30mM Tris-HCl of pH 7.6 and 2% sodium N-lauroylsarcosine) containing 1% proteinase K (Merck, Darmstadt, Germany), and 1% 2-mercaptoethanol. After overnight digestion at 55°C, 0.3M sodium acetate (pH 5.2), 50% Roti-Aqua-Phenol (Roth, Karlsruhe, Germany) and 20% chloroform were added for RNA precipitation. After phase separation through centrifugation, the aqueous phase was carefully removed and added to 2-propanol with 0.02% glycogen (Roche, Basel, Switzerland) as precipitation carrier.

### 3.4. Quantitative real-time real-time polymerase chain reaction

cDNA was synthesized using the High Capacity cDNA reverse transcription kit (Applied Biosystems, Foster City, CA, USA) following the manufacturer’s protocol. cDNA was pre-amplified in 14 PCR cycles with target gene specific primers, thus increasing the sensitivity of the subsequent real-time polymerase chain reaction (RT-PCR) analysis several thousand fold (decrease of CT: 14 cycles; TaqMan PreAmp master mix kit (Applied Biosystems, Foster City, CA, USA), as recommended by the manufacturer. 5 µL cDNA was used in a 20 µL reaction that included 10 µL of Taqman Universal PCR Master Mix (Applied Biosystems, Foster City, CA, USA) and 1 µL of the corresponding Taqman Gene Expression Assay (including primer pair and FAM dye-labeled probe; POLR2A: Hs00172187_m1, ATG5: Hs00355492_m1, BECLIN1: Hs01007019_m1; GABARAPL1: Hs00740588_mH Applied Biosystems, Foster City, CA, USA). The RT-PCR reactions were performed using a Roche Lightcycler 480 System. CT values were calculated by normalization to endogenous control POLR2A and were converted into 2-∆CT values using Excel 10.

### 3.5. Immunohistochemistry

Four micrometer sections of paraffin-embedded biopsies were de-paraffinized and boiled in a citrate buffer for p62 staining and Tris-EDTA buffer for LC3 staining for 20 minutes at 100°C for antigen-retrieval, followed by blocking of endogenous peroxidase activity by 3% H2O2, subsequently blocked with 5% milk and incubated overnight with anti-p62 primary antibody (1:200, p0067, Sigma-Aldrich, St Louis, MO, USA), anti-LC3 primary antibody (2.5 μg/mL, 5F10; Nanotools, Teningen, Germany) at 4°C. Staining was visualized using the ABC Vectastain kit (Vector Laboratories, Burlingame, CA, USA) and sections were counterstained with haemotoxylin (Fluka analytical, Deisendorf, Germany). A blinded expert in renal histopathology identified podocytes and scored them from 1 to 5 on the basis of p62 staining intensity where 5 represents the maximal intensity. For LC3 stained sections, glomeruli pictures were taken at 1000X magnification and the staining (brown colour) was quantified as area fraction normalized to area of glomeruli using ImageJ software.

### 3.6. Ethical issues

1) The research followed the tenets of the Declaration of Helsinki; 2) informed consent was obtained, and 3) the research was approved by the ethical committee of Hannover Medical School.

### 3.7. Statistical analysis

Results are expressed as means ± SD. Statistical significance was determined by Mann-Whitney U test (GraphPad Prism software, San Diego, CA, USA). *P* < 0.05 was considered to be statistically significant.

## 4. Results

### 4.1. Quantification of autophagosomes by transmission electron microscopy

**Figure 1:**
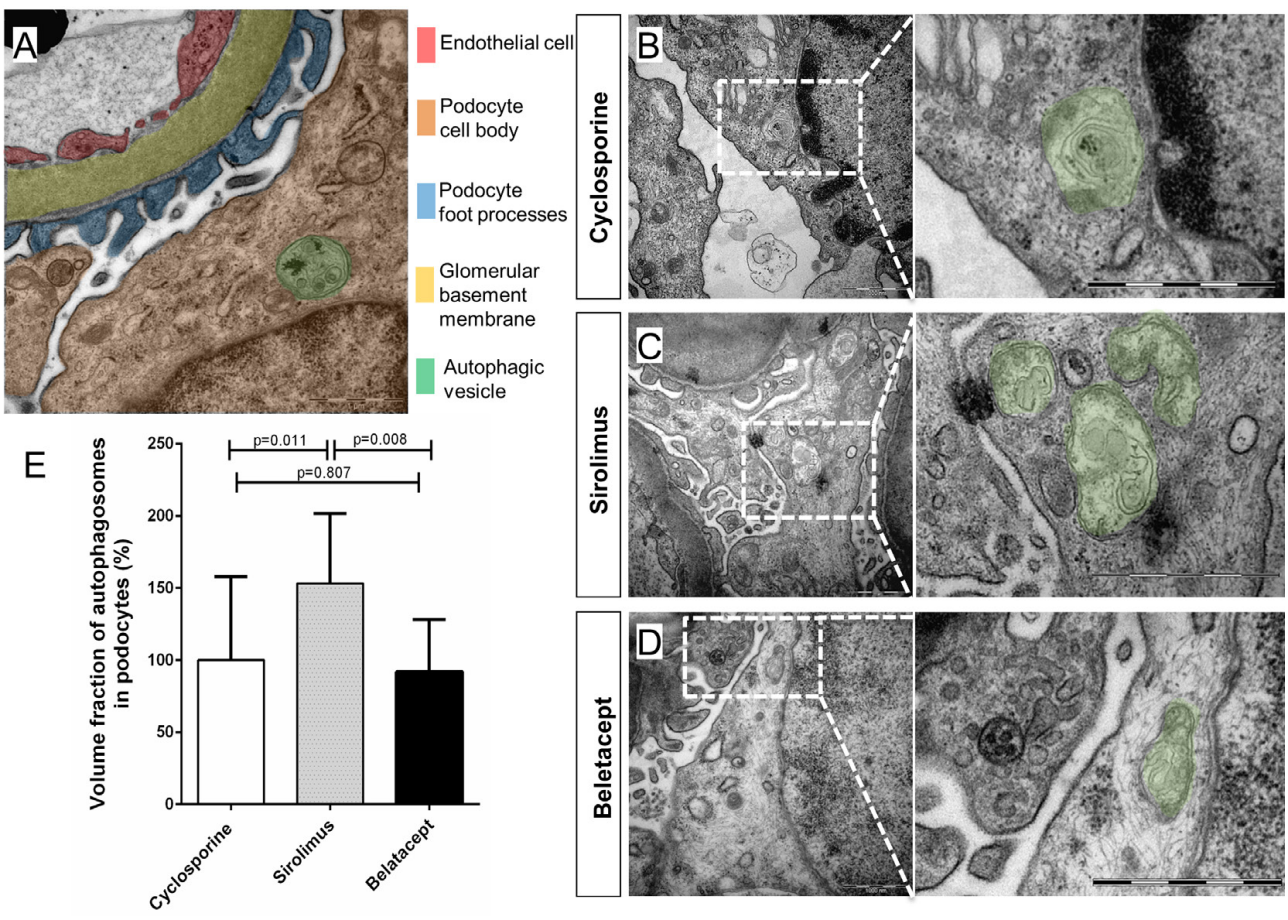
A-E, Quantification of autophagosomes by transmission electron microscopy (TEM) in renal allograft biopsies. A, TEM showing ultrastructural features of colour coded glomerular cells with endothelium in red, glomerular basement membrane in yellow, podocyte cell bodies in brown and podocyte foot processes in blue. Note the autophagic vesicle in the podocyte cell body (green). B-D, Representative TEM ultrastructural images of autophagic vesicles in cyclosporine, sirolimus and belatacept treated patients. E, Sirolimus treatment is associated with a significantly higher autophagosomal volume fraction as compared to cyclosporine treatment and belatacept treatment. All values are mean ± SD; n = 20 for cyclosporine, n = 15 for sirolimus and n = 6 for belatacept. Original magnification in A-D 22000X: scale bar = 1 μm.

By virtue of its high resolution, TEM can be used to visualize autophagosomes directly in tissue samples (7). Transplant biopsies were compared using TEM between sirolimus and cyclosporine treated patients. Clinical characteristics are summarized in Table 1. In order to reach optimal intersample comparability we specifically restricted our analysis on podocytes; a homogenous cell type that can be unambiguously identified by TEM and is known for its strong autophagic involvement (10-12). Pilot studies revealed that the analysis of other renal cell types, e.g. tubular cells, introduced a strong selection bias as they comprise different subtypes which possess individual rates of baseline autophagy (12). Autophagosomes were found in podocytes of all patients (Figure 1B-D) but morphometric stereological quantification indicated significantly higher autophagosomal volume fraction in the sirolimus treated group (Figure 1E). Since cyclosporine has also been linked to autophagy induction in some experimental and clinical studies (13), podocytic autophagosomes were also quantified in transplant biopsies from a small cohort of belatacept treated recipients. The mean podocytic autophagosomal volume fraction in biopsies of these patients, who were CNI free was similar to the cyclosporine treatment group and significantly lower than in sirolimus treated patients (Figure 1E ). These findings indicate that sirolimus treatment increases the load of autophagosomes in podocytes.

**Table 1:** Patients’ characteristics

**Factor**	**Sirolimus (n = 15)**	**Cyclosporine (n = 20)**	**Belatacept (n = 6)**
Recipient age (y)	51 ± 13	44 ± 14	53± 15
Male sex (n)	12	13	4
eGFR (mL/min)	58 ± 27	67 ± 28	62 ± 8
Sirolimus plasma concentration (ng/mL)	8.7 ± 5	-	-
Daily dosage of sirolimus (mg/d)	2.3 ± 1.8	-	-

Abbreviation: eGFR, estimated glomerular filtration rate.

### 4.2. Assessment of autophagy by expression analysis and immunohistochemistry

**Figure 2:**
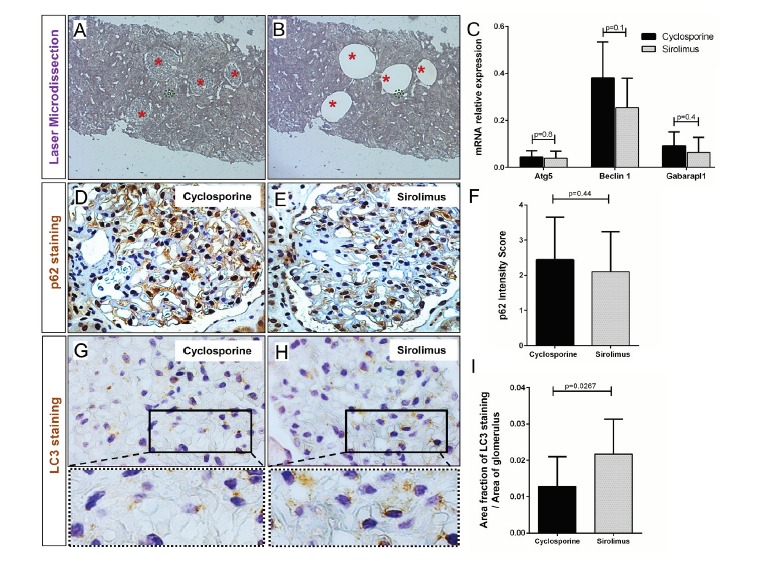
A-C, Quantification of autophagy on the transcriptional level using laser microdissected glomeruli from renal allograft biopsies. Representative images of paraffin sections before and after laser microdissection (glomeruli marked by asterisks, A, B). Quantitative RT-PCR from microdissected glomeruli for Atg5, Beclin-1 and Gabarapl1 mRNA (C). D-F, Immunohistochemistry of p62 in renal allograft biopsies. D, E, Representative immunohistochemical staining of p62 in glomeruli. F, Semiquantitative scoring of podocyte staining intensity (1 to 5; 5 representing maximum intensity). G,H, Representative immunohistochemical staining of LC3 in glomeruli. I, Quantitative analysis of LC3 stained area. All values are mean ± SD; n = 11 for C and F. Original magnification in A,B = 200X; in D,E = 400X and in G,H = 1000X.

While the rapid activation of autophagy is thought to occur mainly via changes in kinase activity, the chronic administration of mTOR inhibitors can induce transcriptional changes of autophagy related genes (14,15). In order to investigate whether podocytic changes seen with TEM were paralleled by changes in gene expression we performed laser capture microdissection of glomeruli (Figure 2A,B), thereby enriching podocytic transcripts for expression analysis by quantitative RT-PCR (16). Expression levels of all tested genes (*Atg5*, *Beclin1* and *Gabarapl1*) showed no discernible differences between the groups (Figure 2C) indicating that changes seen in TEM were not paralleled by increased transcription of autophagy pathway genes. Biopsy paraffin sections from the same patients were used for immunohistological analysis of p62. p62 is a typical autophagy substrate which accumulates when autophagic activity is low (1). p62 immunoreactivity was evaluated using a semiquantitative scoring system for podocyte staining intensity. There was a trend for less p62 in sirolimus treated patients but no statistically significant differences were detected (Figure 2D-F). Another important hallmark of autophagy is the formation of cellular autophagosomal punctae containing light chain 3 (LC3). Assessment of immunhistochemical LC3 staining revealed a significantly increased area fraction of granular LC3 positivity in glomeruli of sirolimus treated patients as compared to cyclosporine controls (Figure 2G-I).

## 5. Discussion

A link between mTOR inhibition and autophagy has so far only been shown in experimental studies. One important reason for the lack of clinical studies might be the limited applicability of autophagy detection methods for human tissues (17,18). In our study we analyzed the impact of mTOR inhibition on autophagy in the clinical setting of renal transplantation.

By stereological TEM analysis we found that sirolimus treatment was associated with a significantly elevated volume fraction of podocytic autophagosomes in routinely performed kidney transplant protocol biopsies. TEM is the gold standard to detect autophagosomes and it is a powerful tool for studying autophagy in mammalian tissues (1). However, TEM is not always available and TEM-based stereological analysis is very labor-intensive and requires well-trained personnel. Thus the use of TEM is limited for larger cohort studies and for clinical routine application. Elaboration of alternative methods for monitoring autophagy in routine paraffin-embedded tissues is still evolving (17). We used laser-dissected glomeruli to analyze transcription of autophagy proteins Atg5, Beclin1 and Gabarapl1 but found no differences between the different treatment groups. This lack in transcriptional changes is consistent with previous biopsy microarray studies in mTOR inhibitor treated patients (19,20). It is important to note, however, that unaltered expression levels can not exclude sirolimus-associated autophagic activation since autophagic activity can be regulated without transcriptional changes. Another important method to monitor autophagy is the conversion of LC3-I to LC3-II which is a crucial part of the initiation process of autophagy and leads to the association of LC3 with the autophagosomal membrane (21). Immunoblot is the gold standard to quantify the conversion of LC3 under experimental conditions and fluorescent microscopy can be used to detect autophagosomal LC3 in vivo if LC3 has been genetically tagged with markers like green fluorescent protein (10). As these methods can not be used for patient biopsy material it has been tried to evaluate LC3 indirectly by immunohistochemistry which yielded heterogeneous results (18,22). Using sensitive immunhistochemical staining we observed an increased area fraction of granular LC3 immunopositivity in glomeruli of sirolimus treated patients. This is in agreement with a recent report in which a novel LC3 reporter mouse revealed that systemic administration of sirolimus leads to significantly increased LC3 activation (12).

Several limitations of our study should be acknowledged. First, as an observational study, only associations, but no cause-and-effect relationships, can be established. This problem might have been aggravated by the small cohort size of our study. As kidney transplant recipients represent a very heterogeneous group with a high load of patient-individual covariates renal transplant studies usually require larger patient numbers (23). However, labour intensity of stereometric TEM analysis prevented a better powered cohort size and the option to perform functional correlation analysis with clinical data. Despite this shortcoming, our EM results unequivocally show that sirolimus treatment is significantly associated with increased podocyte autophagosomal volume fractions. It is important to note that our data can not provide an unequivocal answer whether this increase represents enhanced autophagic flux. It could also result from blockade of downstream steps in the autophagic pathway. Normally, lysosomes are restored from autophagosomes in a process that has been described as autophagic lysosomal reformation (24). Failure of this process can disrupt the autophagic equilibrium leading to a cellular build-up of autophagolysosomes which cannot be distinguished from earlier autophagosomes by TEM (25). Therefore, our results might reflect altered autophagosomal cycling rather than enhanced autophagic flux which could explain unaltered gene expression of *Atg5*, *Beclin1* and *Gabarapl1* and unchanged immunostaining for p62 which is an indicator of autophagic degradation. In that case, the intensified LC3 immunostaining might correspond to an autophagosomal buildup without reflecting changes in the net autophagic process of substrate degradation.

## 6. Conclusions

In summary, our TEM data focusing on podocytes in kidney transplant biopsies of patients who received sirolimus as part of their routine care show an increased relative volume density of autophagosomal vesicles. This indicates for the first time an association of sirolimus treatment and autophagosomal processing in the clinical setting of transplantation. Parallel biochemical findings suggest that mTOR inhibition might induce autophagosomal buildup rather than increased autophagic flux. Further research is needed to investigate potential functional consequences on short- and long-term outcome in patients treated with mTOR inhibitors.

## Limitations of the study

This is a retrospective single-center study with a limited number of patients. Biochemical monitoring of autophagic flux was not possible since archived biopsy samples were used.

## Authors’ contribution

RS and AM designed the project. CW, JH and MO planned the EM studies and provided technical EM support. SB,TDK and AB performed the experiments and collected the data. J-HB, CB, WG, HH and JUB provided patient material, clinical data and technical advice. SB, RS, AB and AM performed the data analysis. SB, AM and RS wrote the manuscript. All authors reviewed and approved the final manuscript.

## Conflicts of Interest

The authors have no conflicts of interest to declare.

## Funding/Support

This article is part of the thesis of SB who was supported by Hannover Biomedical Research School (HBRS). The study was supported by the Deutsche Forschungsgemeinschaft (Sonderforschungsbereich SFB 738 to AM and RS).
